# Inattention, Impulsivity, and Hyperactivity in Deaf Children Are Not Due to Deficits in Inhibitory Control, but May Reflect an Adaptive Strategy

**DOI:** 10.3389/fpsyg.2021.629032

**Published:** 2021-02-12

**Authors:** María Teresa Daza González, Jessica Phillips-Silver, Remedios López Liria, Nahuel Gioiosa Maurno, Laura Fernández García, Pamela Ruiz-Castañeda

**Affiliations:** ^1^Department of Psychology, University of Almería, Almería, Spain; ^2^Center for Neuropsychological Assessment and Rehabilitation (CERNEP), University of Almería, Almería, Spain; ^3^Growing Brains, Washington, DC, United States; ^4^Department of Nursing, Physiotherapy and Medicine, University of Almería, Almería, Spain

**Keywords:** deaf children, attention deficit hyperactivity disorder, conduct disorder, inhibitory control, inattention

## Abstract

The present study had two main aims: (1) to determine whether deaf children show higher rates of key behaviors of ADHD (inattentive, hyperactive, and impulsive behaviors) and of Conduct Disorder—CD—(disruptive, aggressive, or antisocial behaviors) than hearing children, also examining whether the frequency of these behaviors in deaf children varied based on cochlear implant (CI) use, type of school (regular vs. specific for deaf) and level of receptive vocabulary; and (2) to determine whether any behavioral differences between deaf and hearing children could be explained by deficits in inhibitory control. We measured behaviors associated with ADHD and CD in 34 deaf and hearing children aged 9–10 years old, using the revised Spanish version of the Conners scale. We then assessed inhibitory control ability using a computerized Stroop task and a short version of the Attention Network Test for children. To obtain a measure of the level of receptive vocabulary of the deaf children we used a Spanish version of the Carolina Picture Vocabulary Test for Deaf and hearing-impaired children. Deaf children showed significantly higher rates of behaviors associated with ADHD and CD, and over 85% of cases detected with high risk of ADHD-inattentive type in the entire present sample were deaf children. Further, in the group of deaf children a negative correlation was found between receptive vocabulary and frequency of disruptive, aggressive, or antisocial behaviors associated with CD. However, inhibitory control scores did not differ between deaf and hearing children. Our results suggested that the ADHD-related behaviors seen in deaf children were not associated with a deficit in inhibitory control, at least in the interference suppression subcomponent. An alternative explanation could be that these behaviors are reflecting an adaptive strategy that permits deaf children to access information from their environment which is not available to them via audition.

## Introduction

Deaf and hard-of-hearing children growing up in hearing communities (hereafter referred to as deaf^1^) have been reported to have elevated rates of behavior problems when compared to typical hearing children of the same age (Barker et al., 2009; Stevenson et al., 2010). Known generally as externalizing behaviors, or negative outward behaviors (Campbell et al., 2000), they include two main groups of behaviors that are reported to be especially high in deaf children. The first group consists of behaviors that reflect cognitive executive functions, specifically inattention, impulsivity and hyperactivity, which are the central features of Attention Deficit Hyperactivity Disorder (ADHD). The second group consists of behaviors that are often considered to have a more emotional origin, specifically disruptive, aggressive or antisocial behaviors. This group of behaviors is frequently characterized as Conduct Disorder (CD), and often coexists with ADHD.

For years researchers have tried to understand the frequency and source of behavioral problems in deaf children, but it is still unclear—especially with respect to inattention, impulsivity and hyperactivity behaviors. For example, disruptive, aggressive, and antisocial behaviors in deaf children have been attributed to the emotional frustration of frequent problems with language and communication, due to having been deprived of full language input during the sensitive period in development—especially in deaf children who do not receive a cochlear implant until a relatively late age (Barker et al., 2009). However, with respect to inattention, impulsivity and hyperactivity behaviors, which reflect cognitive executive functions, there is no clear argument.

Although some previous studies have suggested that children with auditory deficits might show a higher propensity for hyperactivity and other characteristic behaviors of ADHD (e.g., Kelly et al., 1993; Hindley and Kroll, 1998), in a more recent meta-analysis, Stevenson et al. (2015) concluded that deaf children did not show increased rates of ADHD relative to typical hearing children. Instead, the authors suggested that the presence of hyperactivity behaviors has been overestimated in prior studies due to the fact that these behaviors can be difficult to assess in deaf children. Nevertheless, it is noteworthy that no prior studies in deaf children—including those reviewed in Stevenson et al. (2015)—have used assessments specifically designed to distinguish between these two groups of behaviors: inattention, impulsivity, and hyperactivity vs. disruptive, aggressive, and antisocial behaviors. In order to determine whether there are differences between deaf and hearing children in the frequency and source of these two groups of behaviors, it is necessary to use evaluation tools that allow us to distinguish between them.

In addition, it is important to consider whether the behaviors with a cognitive basis (inattention, impulsivity, and hyperactivity) in deaf children are specifically associated with language impairments, as well as with executive function impairments. As described above, prior studies that have attempted to show a relationship between inattention, impulsivity and hyperactivity behaviors and auditory deficits are problematic, and may misattribute the source. It is important to note that deaf children experience a great variability in the extent of auditory or language deprivation and related deficits, depending on the type of deafness or hearing loss, and the use of hearing aids and cochlear implants. In fact, recent research has shown that it is the extent of language deprivation, and not auditory deprivation, that determines whether a deaf child will experience impairments in cognitive executive functions (Hall et al., 2017).

A recent study examined deaf children between ages 6 and 16 years who had received CIs at an early age (age at implantation 2.7 ± 1.9; 45% of children implanted in one ear only and 55% bilaterally) (Boerrigter et al., 2019). This study measured the frequency of externalizing behaviors reported by teachers and parents through the Child Behavior Checklist and the Teacher Report Form, and found that they were similar to those of hearing children. However, among deaf children, the authors found a significantly higher frequency of externalizing behaviors in children with CIs who scored lower in speech perception and receptive vocabulary, compared with those with higher scores (Boerrigter et al., 2019). According to Boerrigter et al. (2019), better oral language perception and production abilities in children who receive CIs at an early age explains why the participants in their study had rates of externalizing behavior problems comparable to those of typical hearing children. Their conclusions are consistent with the idea that receptive language ability is related to cognitive executive function ability.

Thus, differences between deaf and hearing children in behavior problems—especially the inattention, impulsivity and hyperactivity that are central to ADHD—seem to be related to language access, although the mechanism for these differences remains unclear. Some authors have suggested that the source of these behaviors in deaf children is impaired executive functions, especially inhibitory control, since this component has been shown to be impaired in hearing children with ADHD and with disruptive behavior disorders (e.g., Pennington and Ozonoff, 1996; Willcutt et al., 2005; Alderson et al., 2007).

Even in preschool children with typical hearing, externalizing behaviors have been associated with deficits in inhibitory control (see Schoemaker et al., 2013, for a review), which is consistent with the idea that the underlying executive function abilities depend upon—or at least develop concurrently with—language development. The relationship between language, inhibitory abilities and brain function is an ongoing topic of investigation (e.g., Morasch and Bell, 2011). Although such studies on deaf children are scarce, it has been suggested that they are even more likely to have deficits in executive functions (e.g., Hintermair, 2013), and this pattern could be related to the data on increased behavior problems.

In contrast with the argument based on executive function ability, some authors have proposed that the inattention, impulsivity, and hyperactivity that deaf children more frequently present in school could actually be a compensatory strategy, which is required to adapt to and obtain information from their environment (e.g., Bosworth and Dobkins, 2002; Parasnis et al., 2003; Oberg and Lukomski, 2011). According to this hypothesis, early auditory deprivation leads to a compensatory reorganization of visual attention resources, enhancing attention to the peripheral visual space. In the context of a typical classroom where visual attention is expected to be uniformly focused, an increased attention to the peripheral space could appear like behaviors that are classified as inattentive or impulsive/hyperactive by teachers.

Some authors have even argued that poorer performance of deaf children on more complex executive problem solving tasks could be due to the fact that such tasks require the children to distribute their attention resources differently than hearing children do, in order to facilitate orientation of attention (Dye et al., 2008). Therefore, previously observed poor performance of deaf children on tasks of executive functions might not be related to deficits in executive functioning *per se*, but rather to the degree of visual orientation abilities.

All of the above raises the question of whether there is actually an impairment in cognitive executive functioning—specifically inhibitory control—which contributes to the elevated rates of externalizing behaviors in deaf children. We designed the present study, which examined children between 9 and 10 years of age in their school environments, with two primary aims. Our first aim was to observe whether the frequency of inattention, impulsivity and hyperactivity behaviors, as well as disruptive, aggressive, and antisocial behaviors, was higher in a deaf group than in a matched hearing group. Within the deaf group, we sought to examine whether the frequency of these behaviors varied based on the following factors: (1) CI use, (2) type of school (a school for deaf children vs. a school for hearing children), and (3) level of receptive vocabulary. The second aim was to determine whether any behavioral differences between deaf and hearing children could be explained by deficits in inhibitory control.

To measure frequency of inattention, impulsivity and hyperactivity, and disruptive, aggressive and antisocial behaviors, we used a revised Spanish version of the Conners scale for teachers, called the EDAH scale (Farré and Narbona, 2003). This questionnaire is completed by the teachers/tutors who spend the most time with the children in the classroom. In addition to quantifying the frequency with which teachers observe problematic behaviors in the classroom, it has also been shown to be a valid and reliable instrument in the educational environment for detecting ADHD in children aged 6–12 years, in any of the three clinical sub-types (inattentive type, impulsive-hyperactive type or combined type). Another advantage of the EDAH scale is that it allows for distinguishing between ADHD and CD and determining whether the latter is a singular diagnosis or is secondary to ADHD.

In order to measure inhibitory control in deaf and hearing groups, two computerized tasks were used: a computerized version of the Stroop task (Fuentes et al., 2003) and a short version of the Child-ANT (Rueda et al., 2004). The Stroop task used in the present study has been shown to be adequate to obtain a Stroop interference effect in 7-year-old hearing children (Fuentes et al., 2003), since it only requires that children have automated reading of three color words (“RED,” “GREEN,” and “BLUE”). The Child-ANT does not use verbal information, only visual information, and the version used in the present study has been shown to be adequate in deaf children from age 6 (Daza and Phillips-Silver, 2013).

Taking into account the previous literature, we expected first that inattention, impulsivity and hyperactivity would be more frequent in deaf than in hearing children. Second, if these behaviors are associated with deficits in an inhibitory control mechanism, we expected deaf children to show significantly lower performance in both conflict tasks compared to hearing children.

## Materials and Methods

### Participants

This study consisted of 34 deaf and hearing children between the ages of 9 and 10 (9.6 ± 0.49) years, without psychiatric or neuropsychological antecedents. Both groups (deaf *n* = 17; hearing *n* = 17) were paired in sex (5 F, 12 M) and age (deaf group mean = 9.6 ± 0.49 years; hearing group mean = 9.5 ± 0.5). Participants were recruited from two public schools and two deaf children's associations from different localities. Seven of the children in the deaf group attended a public school for deaf children, while the other 10 attended typical public schools but belonged to an association for deaf children.

All deaf children were pre-lingually deaf (loss of hearing was diagnosed before age 2), and of hearing parents. The amount of hearing loss was estimated using the participants' latest audiogram. The degree of hearing loss was defined by unaided hearing in the better ear as (Clark, 1981): Mild (<40 dB); Moderate (41–70 dB); Severe (71–90 dB); or Profound (>90 dB). The severity of hearing loss was distributed thus: moderate, *N* = 4 children; severe, *N* = 3 children; and profound or cofosis, *N* = 10 children. The type of deafness according to lesion localization was neurosensorial in 11 children, unknown in 5 children, and one of transmission (the outer or middle ear). The cause of deafness was prenatal and congenital in 9 children and unknown in the rest.

Nine of the 17 deaf children had a cochlear implant in one ear only (age at implantation = 4.0 ± 1.8 years). Regarding preferred mode of communication, 10 children used oral language, 5 used oral language with the support of Spanish Sign Language (SSL), and two 2 used SSL exclusively.

### Assessment

#### Evaluation of ADHD

The EDHA scale (Farré and Narbona, 2003) provides a measure of inattention (or attention deficit), impulsive, or hyperactive behaviors associated with ADHD, in addition to disruptive, aggressive, or antisocial behaviors that characterize CD, and which can coexist with ADHD. The scale consists of a total of 20 items and must be completed by the child's teacher. Each item describes a behavior of inattention (e.g., “*Spaces out, oblivious*”), impulsivity or hyperactivity (e.g., “*moving constantly, restless*”), or a disruptive, aggressive or antisocial behavior associated with CD (e.g., “*argues or fights over anything*”). These 20 items are grouped into three subscales: (1) 5 items for the inattention or attention deficit subscale, (2) 5 items for the impulsivity/hyperactivity subscale, and (3) 10 items for the CD subscale. Each item is scored from 0 to 3 according to the frequency of the behavior perceived by the teacher (Never = 0; Sometimes = 1; Often = 2; Very often = 3). The raw score obtained in each subscale can be compared with normative scores to obtain a centile score. Furthermore, the scale includes a classification system with two cutoff points based on several statistic and epidemiological values, marking high and low risk zones for ADHD (No risk between 0 and 85 centile; moderate risk between 90 and 94; and high risk between 95 and 100). This scale was validated with a sample of 2,400 hearing children between 6 and 12 years of age and a reliability index (Cronbach's alpha coefficient) equal to or higher than 0.9 (Farré and Narbona, 2003).

### Receptive Vocabulary

To obtain a measure of the level of receptive vocabulary of the deaf children we used a Spanish online version of the Carolina Picture Vocabulary Test for Deaf and hearing-impaired children (CPVT; Layton and Holmes, 1985). The test consists of 130 trials in which the child must indicate which of four drawings (of objects, actions) that appear on the screen correspond with the name that the examiner pronounced orally or signed (in accordance with the preferred communication mode of each child). If the child commits six consecutive errors, the test ends.

#### Executive Functions

##### Computerized Version of the Stroop *task*

To resolve the conflict experienced in the computerized Stroop task (Fuentes et al., 2003), subjects must overcome the powerful tendency to read the word in favor of responding to the color dimension. When subjects must indicate the color of incongruent words (e.g., the word RED in blue letters), difficulty ignoring the intrusive effects of the words results in worse performance (longer reaction time, and/or more errors) than in a neutral condition in which participants indicate the color of meaningless stimuli, in this case a string of colored Xs. In this task, the conflict resolution score (the Stroop interference effect), is obtained by comparing the performance in the incongruent condition against the neutral condition.

All stimuli were displayed on a laptop computer screen controlled by E-prime 2.0 (Psychology Software Tools, Inc., Pittsburgh, PA; Schneider et al., 2002). Targets were shown the words “ROJO” (red), “AZUL” (blue), and “VERDE” (green) and a row of four Xs, displayed in red, blue, or green color. All stimuli were displayed in uppercase characters against a black background, and they were centered both horizontally and vertically at a viewing distance of ~65 cm. Each trial began with a fixation period of 500 ms and then the colored stimulus (a word in the incongruent condition or a row of four “Xs” in the neutral condition) was displayed until the subject responded. Participants indicated the color of the target by pressing a computer key marked with a blue, red or green sticker, over 72 trials lasting ~10 min. The first 12 were practice trials and were not included in the data analysis. The remaining 60 were experimental trials, 30 for the neutral condition (10 trials per color), and 30 for the incongruent condition (10 trials per color). Children were told that their task would be to indicate the color of the stimulus displayed in the center of the screen by pressing the corresponding colored sticker on the computer keyboard as quickly as possible. They were first shown displays of the different target stimuli and were asked to demonstrate which key on the keyboard they had to press. When words were displayed (incongruent trials), participants were encouraged to focus only on the color of the target word and ignore its meaning. When it was clear that they understood the instructions, children began the practice block.

### Short Version of the Child-ANT

The short version of the Child-ANT (Rueda et al., 2004) used in the present study was created using E-Prime version 2.0. (Psychology Software Tools, Inc., Pittsburgh, PA; Schneider et al., 2002). All stimuli were displayed on a laptop computer screen, viewed from a distance of about 55–60 cm. The Child-ANT uses visual stimuli to separately assess the attentional networks and allows to obtain performance scores of alerting, orienting, and inhibitory control. In the present study, we used only the flanker task (Eriksen and Eriksen, 1974) to assess inhibitory control (through flank interference effect). The task is presented in the form of a computerized game where the object is to “feed” a hungry fish as quickly as possible each time it appears on the screen.

The procedure followed that of Rueda et al. (2004). Each trial began with a fixation cross at the center of the screen for a short, variable period of time (between 400 and 1,600 ms). A cue then appeared in the form of a briefly presented (150 ms) asterisk, followed by a 450 ms pause during which the fixation cross was again visible. The target stimulus (a central fish) was then presented, either above or below the fixation cross. The subject's task was to indicate with a button press whether the target stimulus (the central fish) was pointing to the left or to the right. The target stimulus remained on the screen until a response was detected, to a maximum of 1,700 ms.

There were four cue types: no cue; a single central cue; a double cue; and a spatial cue (presented at one of the possible stimulus locations). Performance with different cue types allows to obtain performance scores of alerting and orienting. Performance with different target stimuli provided a measure of subjects' ability to overcome conflict which was used as an index of inhibitory control capacity. The target stimulus (the central fish) could be flanked by fishes pointing in the opposite direction (incongruent), the same direction (congruent), or could be alone (neutral). Each fish stimulus subtended 1.6° of visual angle and the contours of adjacent fish were separated by 0.21°. The entire stimulus (target fish plus four flankers) subtended a total of 8.84°. The target was presented either about 1° above or below fixation.

The original Child ANT developed by Rueda et al. (2004) consisted of 24 practice trials and 3 experimental blocks of 48 trials in each, lasting ~30 min. In the short version of the Child-ANT used in the present experiment, the number of experimental blocks were decreased from three blocks (Rueda et al., 2004) to one block. This reduction was made in order to prevent the children would become overly fatigued or bored completing 30 min of the same task. With this reduction, the Child ANT lasting ~10 min (duration similar to that of the Stroop task). Each child was tested individually with the experimenter present throughout the test.

Instructions were given to the participants in their preferred mode of communication by a research assistant fluent in spoken Spanish and SSL. Children were told that a hungry fish would appear on the screen and they must feed the fish by pressing the button on the mouse that matched the way the fish was swimming. They were first shown index displays of the single rightward and leftward fish stimuli (corresponding to the neutral condition) and were asked to demonstrate which button on the mouse would successfully feed the fish. They were then told that sometimes the hungry fish would be alone, the way they had just seen, and sometimes the fish would be swimming with some other fish as well. They were instructed that in this case they should pay attention to the fish in the middle and feed that fish using the mouse. The experimenters then showed the participants displays showing the stimuli in a congruent configuration and an incongruent configuration and asked them to demonstrate which button they should press to feed the fish in the middle. Finally, participants were instructed to maintain fixation on the cross in the center of the screen throughout the task and to respond as quickly and accurately as possible. When it was clear that they understood the instructions, children began the practice block. After each response, the participant received visual feedback from the computer. For correct responses, a simple animation sequence showed the target fish blowing bubbles. Incorrect responses were not followed by the animation of the fish.

### Procedure

Authorization to conduct this research was obtained by the bioethical committee of the University and signed informed consent from the parents of participating children.

The EDAH Scale was completed by the children's teachers for each participant in the deaf and hearing groups. Each child completed the two tasks (Child-ANT and Stroop) individually in a single session of 20 min. For deaf children, instructions were given in their preferred communication system (oral or SSL). For the deaf group, the receptive vocabulary test was administered in a subsequent experimental session, 1 week later. All children were tested in a quiet room of the school or association to which they belonged.

### Statistical Analysis

All statistical analyses were conducted with the last version of the statistical program SPSS. First, to calculate if the variable followed a normal distribution Kolmogorov–Smirnov and Shapiro–Wilk tests were used. For comparisons between groups (deaf vs. hearing, deaf without IC vs. deaf with IC), the parametric Student *t*-test was used for groups with normal distribution while the non-parametric Mann–Whitney test was used for groups without normal distribution. We used two-tailed tests because before setting up the experiment and running the test, we expect that if a difference between the groups is highlighted, we did not really know the sign of the potential difference. We used the Spearman Rho statistic for the analysis of correlation between the centile scores obtained in the three sub-scales of the EDHA and the scores obtained in the receptive vocabulary test.

## Results

### Differences Between Deaf and Hearing Groups in Externalizing Behaviors

**Figure 1:**
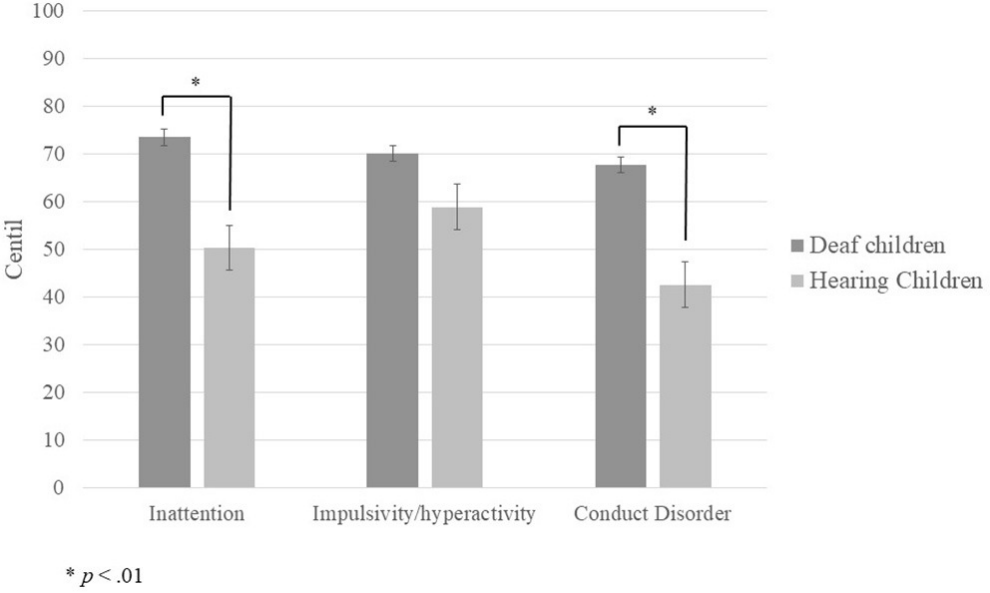
Mean centile scores obtained by deaf and hearing children in each of the EDAH sub-scales.

To determine differences in externalizing behaviors between children groups, each child's raw score from each EDAH subscale was first compared with a normative scale to obtain a centile. The results of the comparison analysis between all deaf and hearing children (see Figure 1) show significant differences in the inattention subscale (*U* = 76; *p* = 0.007) and conduct disorder subscale (*U* = 38; *p* = 0.000), and a marginally significant difference in impulsivity/hyperactivity (*t* = −1.787; *p* = 0.08).

**Figure 2:**
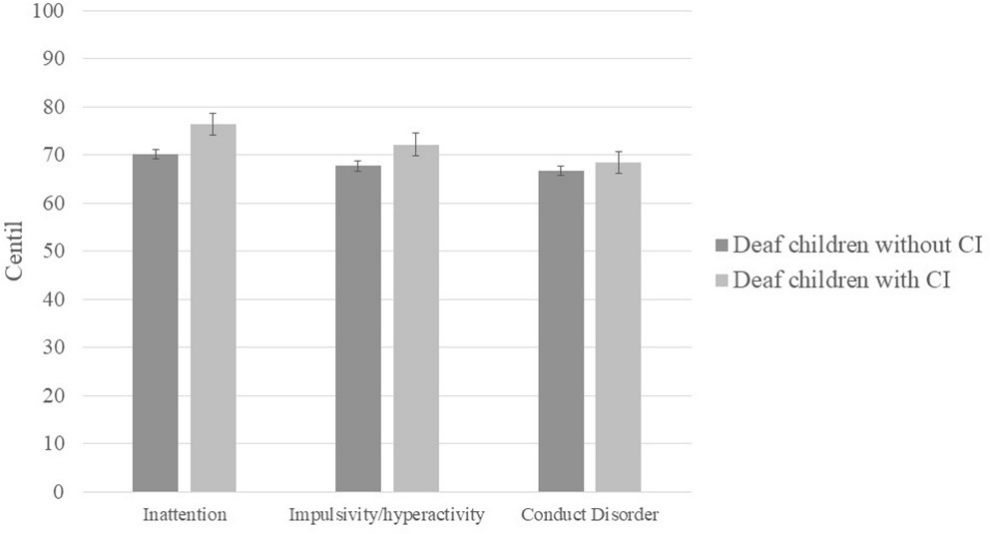
Mean centile scores obtained by deaf children with and without CI in each of the EDAH sub-scales.

**Figure 3:**
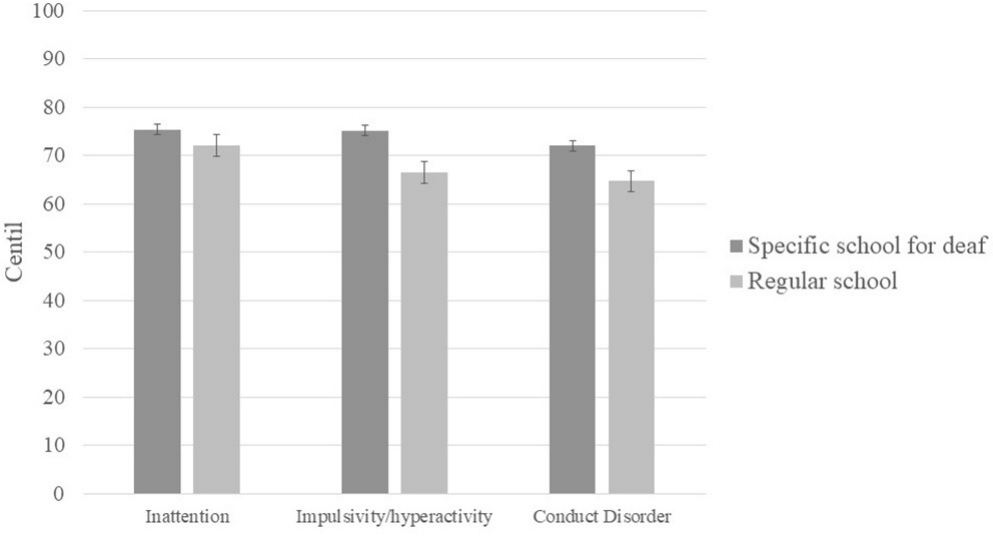
Mean centile scores obtained by deaf children attending a specific school for the deaf and children attending a regular school.

Next, we compared the centile scores of deaf children without CIs (*n* = 8) to those with CIs (*n* = 9), and found no significant difference on any EDAH subscale (inattention: *U* = 32; *p* = 0.69; impulsivity/hyperactivity: *t* = −0.478; *p* = 0.64; conduct disorder: *U* = 33.5; *p* = 0.81; see Figure 2). We also found no significant difference in any of the sub-scales of the EDAH as a function of whether children attended a school for deaf children or a typical public school (inattention: *U* = 29.5; *p* = 0.59; impulsivity/hyperactivity: *t* = 0.945; *p* = 0.36; conduct disorder: *t* = 0.767; *p* = 0.455; see Figure 3).

In accordance with the classification system of the EDAH scale to detect the risk of the three types of ADHD and CD associated with ADHD as proposed in the DSM-IV, a percentage of children with moderate-to-high risk (a score of over 90%) was estimated for each of the clinical subtypes (Table 1). For all clinical subtypes of ADHD and CD, the risk was higher in the deaf group, although only the inattention type was marginally significant (*p* = 0.08).

**Table 1:** Cases with risk of ADHD and CD detected by EDAH.

	**Total high risk cases**	**% of the total cases of risk in DEAF group**	**% of the total cases of risk in hearing group**	**Statistic**	***p*-value**
Inattention ADHD	7	85.7	14.2	Fisher's exact test	0.08
Impulsive/Hyperactive ADHD	6	66.6	33.3	Fisher's exact test	0.65
Mixed ADHD	7	71.4	28.5	Fisher's exact test	0.39
Conduct Disorder	3	100	0	Fisher's exact test	0.22

### Relation Between Externalizing Behaviors and Receptive Vocabulary in Deaf Children

The correlational analysis between the centile scores obtained from the sub-scales of the EDAH and the scores on receptive vocabulary resulted in a significant negative correlation only between disruptive, aggressive and antisocial behaviors and receptive vocabulary (see Table 2).

**Table 2:** Correlations coefficients between the centile scores obtained from the three sub-scales of the EDAH and the scores on receptive vocabulary (Spearman Rho).

**Sub-scale EDAH**	**Receptive Vocabulary**
Inattention	−0.25
Impulsivity/hyperactivity	−0.35
Conduct disorder (disruptive, aggressive or antisocial behaviors)	−0.55^*^

* *p < 0.05*.

### Differences in Inhibitory Control Between Deaf and Hearing Children

To obtain inhibitory control scores in the Stroop and Child-ANT tasks, an interference effect was estimated with the measures of speed (reaction time) and precision (percentage of errors). In the Stroop task incongruent trials were subtracted from neutral ones, and in the Child-ANT, reaction times, and percentage of errors of incongruent trials were subtracted from congruent trials (see Table 3).

**Table 3:** Results of inhibitory control tasks.

	**Deaf children**	**Hearing children**	***t*-statistic**	***p*-value**
	***n* = 17**	***n* = 17**		
**Stroop task**				
RT total (in ms)	1,010	979	*t* = −0.564	0.58
% Errors total	3	4.7	*U* = 107.5	0.20
Interference (in ms)	65	100	*U* = 91	0.07
Interference (with errors)	−0.3	1.6	*U* = 121.5	0.43
**ANT-Child**				
RT total (in ms)	736	729	*U* = 143	0.97
% Errors total	4.9	2.2	*U* = 102	0.15
Interference (in ms)	47	82	*U* = 118	0.37
Interference (with errors	2.6	0.4	*U* = 134.5	0.73

Comparing the interference effect between deaf and hearing children in the Stroop and ANT-Child tasks, only a marginally significant difference was found for reaction time (RT) in the Stroop task (*p* = 0.07). Surprisingly, deaf children showed a smaller interference effect (65 ms) than hearing children (100 ms). RTs for neutral and incongruent trials were then compared between deaf and hearing children, to determine whether the smaller Stroop interference in deaf children was due to a faster response to incongruent trials or a slower response in neutral trials. As seen in Table 4, deaf children's responses were slower than hearing children's in neutral and incongruent trials, but those differences were not statistically significant. This result suggests that the minor interference effect shown by deaf children in the Stroop task did not reflect greater inhibitory control for resolving the conflict in incongruent trials.

**Table 4:** Mean reaction times obtained by deaf and hearing children on two types of Stroop trials.

	**Deaf children *n* = 17**	**Hearing children *n* = 17**	**Difference between groups**	***t*-statistic**	***p-*value**
**Stroop task**					
Incongruent trials (in ms)	1,042	1,028	14	*t* = −0.213	0.83
Neutral trials (in ms)	977	928	49	*t* = −0.952	0.35

## Discussion

The results of the present study show that in the context of school, teachers report that inattention, impulsivity, and hyperactivity as well as disruptive, aggressive and antisocial behaviors are more frequent in deaf children than in hearing children. These reports are especially frequent for inattention.

This frequency of disruptive, aggressive, and antisocial behaviors is consistent with those of previous studies on behaviors associated with CD (e.g., Vostanis et al., 1997; Van Eldik et al., 2004) and with the results of the meta-analysis of Stevenson et al. (2015). However, with respect to inattention, impulsivity, and hyperactivity behaviors associated with ADHD, the present results differ from the findings of Stevenson et al. (2015) These authors reviewed 12 studies which used the “Strengths and Difficulties Questionnaire,” and concluded that there was no indication that deaf children had a specific propensity to develop hyperactivity. It is important to note however that this questionnaire is a screening tool for psychopathologies in children and adolescents, and not a specific evaluation for inattention, impulsivity, and hyperactivity behaviors.

Our results showed significantly higher rates especially of inattention behaviors in deaf than hearing children. In addition, of all the cases of moderate-high risk of ADHD-inattentive type that were detected in the present sample of participants (7 of 34 children), 85.7% of those cases were deaf children. In the group of deaf children, 35.2% were determined to have a moderate to high risk of ADHD-inattentive type, while in the hearing group that rate was 5.8%. However, this sample size is small and therefore the results should be interpreted with caution.

It is important to consider whether the present study overestimates inattention, impulsivity and hyperactivity behaviors, as Stevenson et al. (2015) suggested, due to difficulties in evaluating deaf children. To this end, the first question to ask is who is evaluating the deaf children. It is possible that hearing teachers who have little training or experience in teaching deaf children would have more difficulty evaluating them, resulting in inflated incidence rates. In the present study, we found no difference in the frequency of these behaviors as a function of whether the children were evaluated by teachers from a typical public school vs. teachers from a school for deaf children. The second question is whether the assessment tool used to measure the frequency of inattention, impulsivity, and hyperactivity here offers an advantage over those used in previous studies. The EDAH scale is a Spanish version of the Conners Teacher Rating Scale-Revised (CTRS; Conners, 1997). The CTRS is the most widely utilized for measuring the symptoms of ADHD across populations, however in practically all of the previous studies with deaf children, the behaviors associated with ADHD were measured using the “Strengths and Difficulties Questionnaire” (Goodman, 1997), which as noted above is not a specific evaluation for inattention, impulsivity, and hyperactivity behaviors.

The EDAH scale used in the present study has been used to detect ADHD in children from 6 to 12 years of age in the school environment. Among the advantages of the EDAH vs. the original CTRS (Conners, 1969), we highlight the following: (1) there are fewer items, so the evaluation is shorter, (2) items were eliminated that reflected emotion-based processes which were less clear, imprecise and had no statistical significance (Farré and Narbona, 1997), (3) it takes into account the distinction proposed in the DSM-IV between the subtypes of ADHD, and (4) it allows for the isolation of ADHD and CD diagnoses, and to determine whether the latter is a singular diagnosis or rather secondary to AHDH (Farré and Narbona, 2003).

With respect to CI use in deaf children, although Boerrigter et al. (2019) observed that CI use was associated with lower rates of behavior problems, we did not find a significant difference between children with and without CI. However, this result could be explained by the age of implantation. In the experiment of Boerrigter et al., children with CIs were implanted at a much younger age (2.7 ± 1.9 years) than the deaf CI users who participated in our study (4.0 ± 1.8 years). Considering that all participants in the present study were deaf children of hearing parents, the duration of language deprivation was much longer. In fact, when we compared the raw mean scores for receptive vocabulary for deaf CI-users (116.7) against those without CI (110.5) there was no significant difference, suggesting that the linguistic abilities of children with and without CI were equivalent in the present study.

In contrast, the negative correlation that we found in deaf children between level of receptive vocabulary and frequency of behaviors associated with CD is consistent with previous studies that have shown that deficiencies in communication contribute to a higher incidence of behavioral problems (e.g., Edwards et al., 2006; Barker et al., 2009; Dammeyer, 2010; Jiménez-Romero, 2015). For example, Barker and colleagues (2009) showed that language deficits contribute indirectly to behavior problems in two ways: interference with comprehension of the demands and needs of others, and difficulty with regulating emotion in ways that affect behavior. In a study by Dammeyer (2010), among 334 deaf Danish children (90 of which were CI users), the prevalence of “psychosocial problems” was 3.7 times higher than in hearing children. The authors nevertheless concluded that when the language level, whether oral or sign language, of the deaf children was sufficiently good, the frequency of psychosocial problems decreased.

Regarding inhibitory control, the present results showed similar levels of ability between deaf and hearing children. In both the Stroop and Child-ANT tasks, deaf children did not show a greater interference effect than hearing children, suggesting that both groups of children were able to recruit mechanisms of inhibitory control to suppress or inhibit interference from distracting stimuli. In the Stroop task, the deaf children showed a smaller interference effect than the hearing, with marginal significance. However, this smaller interference effect was not due to faster response times on incongruent trials. As shown in Table 4, reaction times in the incongruent trials were very similar in deaf children (1,042 ms) and hearing children (1,028 ms). On the contrary, in the neutral trials the difference between deaf (977 ms) and hearing (928 ms) children was greater, although this difference did not reach statistical significance either. However, this pattern of results leads us to think that this lower interference effect shown by deaf children does not seem to be reflecting a greater ability to resolve the conflict in incongruent trials, but rather a reduction in speed in neutral trials. Since these differences in neutral trials did not reach statistical significance, we cannot conclude that there is a problem in processing speed in deaf children. However, future studies might explore whether deaf children show typical patterns of processing speed.

Overall, the results in the present study suggest that the higher frequency of ADHD-associated behaviors (especially inattention) is not due to problems with inhibitory control, despite the fact that this component of executive functioning has been found to be altered in children diagnosed with ADHD. It is important to point out that in the past several years there has been a shift in research on inhibitory control (Gandolfi et al., 2014). An increasing number of researchers have supported the idea that inhibitory control is not a unitary construct, but rather a set of functions (e.g., Dempster, 1992; Nigg, 2000; Friedman and Miyake, 2004; Diamond, 2013) among which at least two can be differentiated: response inhibition and interference suppression. The first refers to the capacity to control impulsive behavior in order to prevent (inhibit) motor and verbal responses. The second function involves working memory and refers to the ability to suppress interfering information. This would be used in situations in which there is relevant and irrelevant information for the task such as in the flanker task or the Stroop task, when the stimulus requiring a response has two dimensions, one relevant (e.g., the color the word appears in) and one irrelevant (the word meaning).

These two components of inhibitory control, response inhibition and interference suppression, have been distinguished in older children (Bunge et al., 2002; Martin-Rhee and Bialystok, 2008). However, studies on inhibitory control in deaf children have only focused on response inhibition (e.g., Figueras et al., 2008; Botting et al., 2016), and have found this measure to be different from hearing children. But to our knowledge no previous studies have found differences between deaf and hearing children on suppression of interference. Our results might suggest that this sub-component of inhibitory control is not related to the behaviors of inattention, impulsivity, and hyperactivity that are more commonly reported in deaf children in the school environment. However, in previous studies it has been found that hearing children with ADHD exhibited deficits in both sub-components of inhibitory control: response inhibition (e.g., Wodka et al., 2007) and interference suppression (e.g., Mullane et al., 2009). Therefore, in deaf children there must be other mechanisms that can help explain these behaviors associated with ADHD.

An alternative explanation for the present results could be that the ADHD-related behaviors that are observed in deaf children are reflecting an adaptive strategy for obtaining information in their environment. Some authors (e.g., Oberg and Lukomski, 2011) have suggested the idea that deaf individuals tend to utilize a strategy of visual codification for receiving information from the environment, which requires them to distribute their attentional resources to their central visual field as well as to the periphery. Deaf individuals have a need to explore a wider visual field than hearing individuals, who can focus their attentional resources on the central field while accessing other information via audition. This need causes deaf individuals to shift their attention in the environment more often than hearing individuals, a behavior which can be perceived by others as inattentive or hyperactive. In the classroom, these shifting behaviors are very likely to be reported by teachers as inattentive (as in, “easily distracted” or “shows scarce attention”), or reported as impulsive or hyperactive (as in, “constantly moving,” or “restless”). Nevertheless, for deaf children these behaviors might serve an adaptive function and be necessary for them not to lose track of what goes on in their environment, which cannot be accessed via hearing. Although in our study we did not find an effect of CI use, a result that would be incongruent with this hypothesis, it is important to note that the deaf CI users who participated in our study were implanted at a late age (4.0 ± 1.8 years). The duration of auditory and therefore language deprivation was long for deaf CI users in our study, which could further necessitate such an adaptive strategy for obtaining information in their environment.

The disruptive behaviors that deaf children exhibit in the classroom may be caused by a hearing and visually inaccessible environment. Providing an environment that is accessible for deaf children involves both simple and complex planning strategies (for example, lighting adjustments to decrease eyestrain, use of strategically placed convex mirrors to increase visual access, use of materials that minimize intrusive noise; Berndsen and Luckner, 2010; Martins and Gaudiot, 2012). Further studies could also test the relationship between the environmental characteristics of the classroom and the frequency of the externalizing behaviors associated with ADHD. Another way of thinking about this is that if the environment is adapted to the child, then the child will not carry the burden of having to further adapt themselves while risking being labeled as deficient.

The interpretation of our findings should be considered in light of several limitations. First, we used a relatively small sample size. Future studies should assess the replicability of the present results, in part because of the relatively small sample size, and even more so because the present results depart from those of previous studies which examined the behaviors associated with ADHD but which did not use measures that specifically addressed those symptoms. We also suggest that future research in this area focus on examining how other central executive processes, such as working memory and cognitive flexibility, are related to the inattention, impulsivity, and hyperactivity behaviors that are associated with ADHD in deaf children. Second, although the responses from teachers of hearing children were compared to those from teachers of deaf children, the number of teachers was small. Third, the frequency of behaviors associated with ADHD and Conduct Disorder were only analyzed in the school context (as reported by teachers). It is important for future studies to examine study the frequency of these behaviors as reported by family members at home.

## Conclusion

The results obtained in the present study have important practical implications for the education of deaf children. First, with regard to the disruptive, aggressive, and antisocial behaviors associated with CD, it is crucial that deaf children from a very early age have a language system that they can acquire naturally and that supports communication skills. Communication skills in deaf children, whether through oral language, sign language, or a combination, play a critical role in social functioning and emotional self-regulation.

Second, given that the inattention, impulsivity and hyperactivity associated with ADHD could be a manifestation of an adaptive strategy that permits deaf children to access information from their environment which is not available to them via audition, it is clearly important that the educational environment of a deaf child be designed with this strategy in mind.

## Data Availability Statement

The raw data supporting the conclusions of this article will be made available by the authors, without undue reservation.

## Ethics Statement

The studies involving human participants were reviewed and approved by Ethics Committee of University of Almería. Written informed consent to participate in this study was provided by the participants' legal guardian/next of kin.

## Author Contributions

MD: conceptualization, methodology, investigation, formal analysis, writing of original draft, and revisions. JP-S: conceptualization, methodology, writing of original draft, revisions. RL: writing of original draft. NG, LF, and PR-C: formal analysis, writing of original draft. All authors contributed to the article and approved the submitted version.

## Conflict of Interest

The authors declare that the research was conducted in the absence of any commercial or financial relationships that could be construed as a potential conflict of interest.

## Footnotes

^1^Note that the present study, and much of the research cited in the present article, refers to deaf and hard-of-hearing individuals who are raised in hearing communities, sometimes using hearing devices, often using oral language and some sign language. This research does not refer to individuals who are culturally Deaf, native signers, or who use sign language exclusively. Although no population is truly homogenous, the differences between these populations have important implications for behavior and brain functioning.
